# Perforated Meckel’s Diverticulum With Dual Gastric and Pancreatic Heterotopia: A Pediatric Case Report

**DOI:** 10.7759/cureus.103888

**Published:** 2026-02-19

**Authors:** Ayesha Razakh, Subia Shakeel, Shubha H V, Suguna B V, Vijaya C

**Affiliations:** 1 Pathology, Sapthagiri Institute of Medical Sciences and Research Centre, Bengaluru, IND

**Keywords:** dual heterotopia, ectopic pancreatic tissue, gastric metaplasia, meckel's diverticulum, pediatric surgery, perforation

## Abstract

Meckel’s diverticulum is a common congenital gastrointestinal anomaly that can lead to life-threatening complications such as perforation. While rare in children, a perforated Meckel’s diverticulum often mimics acute appendicitis, posing a significant diagnostic challenge. We report the case of a five-year-old male who presented with acute right lower quadrant pain, hematochezia, vomiting, and signs of peritonitis. Emergency laparotomy was performed, which confirmed a perforated Meckel’s diverticulum densely adherent to the ascending colon. Histopathological examination of the resected specimen revealed the presence of dual heterotopic tissue, consisting of both gastric and pancreatic mucosa, with transmural necrosis at the site of perforation. The patient had an uneventful postoperative recovery. This case underscores the necessity of considering Meckel’s diverticulum in the differential diagnosis of a pediatric acute abdomen, especially when atypical symptoms like rectal bleeding are present. It further highlights the role of histopathology in identifying dual heterotopia as a predisposing factor for perforation.

## Introduction

Meckel’s diverticulum (MD), the most common congenital anomaly of the gastrointestinal tract, has an incidence of approximately 2% in the general population [1-4]. It arises from the antimesenteric border of the ileum due to the incomplete obliteration of the vitelline duct during fetal development. While the majority remain asymptomatic throughout life, symptomatic cases most commonly present in children under the age of 10, with complications occurring in roughly 4% of affected individuals, which can include hemorrhage, intestinal obstruction, diverticulitis, or perforation. These complications often mimic common abdominal emergencies like acute appendicitis, which can create diagnostic challenges in pediatric acute abdomen [5]. Among these complications, perforation is exceptionally rare, occurring in only 0.5% of symptomatic cases; it therefore warrants careful clinical consideration [6].

The presence of heterotopic mucosa is a key determinant of the development of these complications in symptomatic cases, which frequently involve gastric mucosa present in 52% of cases and pancreatic tissue found in only 5% of cases in the pediatric population [7]. While heterotopic gastric mucosa is common, the coexistence of both tissue types is very rare and significantly increases the risk of complications. This coexistence is clinically significant as the combined secretion of acid and proteolytic enzymes creates a synergistic effect that accelerates mucosal damage, leading to early perforation. The learning objective of this case is to highlight this rare dual-tissue mechanism as a predisposing factor for life-threatening complications in the pediatric population. Here, we report a rare case of a perforated Meckel’s diverticulum in a five-year-old male child, complicated by the presence of dual heterotopic tissue (gastric and pancreatic), identified on histopathology. This case highlights the diagnostic challenge of MD in pediatric age groups and underscores the importance of histopathological evaluation in understanding disease mechanisms and guiding clinical insight.

## Case presentation

Clinical history and examination

A previously healthy five-year-old male child presented to the emergency department with a three-day history of acute-onset, progressive abdominal pain. The pain was diffuse, initially periumbilical, constant, and non-radiating, with a reported severity score of 6/10. Over the subsequent 24 hours, the pain localized to the right lower quadrant. The child became increasingly irritable and lethargic, with five to six episodes of recurrent, non-bilious, non-projectile watery vomiting. On the day of admission, the parents reported episodes of hematochezia (fresh blood mixed with stool). The child’s appetite was markedly decreased, and oral intake had been minimal for 24 hours. Notably, he had experienced similar complaints one month prior, which were treated conservatively. There was no significant past medical or surgical history; developmental milestones and immunizations were up to date.

On admission, the child appeared irritable and pale. Physical examination revealed tachycardia (heart rate: 120 beats/min), blood pressure of 90/60 mmHg, a respiratory rate of 22 breaths/min, and a tympanic temperature of 37.8°C. Abdominal examination demonstrated generalized tenderness, maximal around the umbilicus and in the right iliac fossa. Both voluntary and involuntary guarding were present, alongside rigidity and rebound tenderness, clinical signs highly suggestive of generalized peritonitis. Bowel sounds were hypoactive, and no masses were palpable. A digital rectal examination was deferred due to patient distress and overt signs of peritonitis. Cardiovascular, respiratory, and neurological examinations were otherwise unremarkable.

Diagnostic assessment

Laboratory investigations on admission revealed significant microcytic anemia, with a hemoglobin of 6.3 g/dL, a red blood cell count of 2.77 million/mm³, and a hematocrit of 20.5%. These findings were consistent with acute blood loss anemia. The total white blood cell count was 9.30 × 10³/μL with a neutrophilic predominance (64%), while the platelet count remained within normal limits at 272 × 10³/μL. A formal report of a contrast-enhanced computed tomography (CECT) scan, performed at the referring secondary center, described a thickened, peripherally enhancing, tubular, fluid-filled structure measuring 5.5 mm in diameter, arising from a small bowel loop in the right subhepatic region. Significant adjacent mesenteric inflammation was noted. The tip of the structure was poorly visualized and terminated in an irregular, hypodense collection adjacent to the hepatic flexure of the colon, suggestive of a contained perforation. Additional findings included circumferential wall thickening of the hepatic flexure, a pulled-up cecum, and multiple enlarged regional lymph nodes. The appendix was visualized and appeared normal. The overall radiological impression was acute Meckel’s diverticulitis with perforation at the tip.

Therapeutic intervention

**Figure 1:**
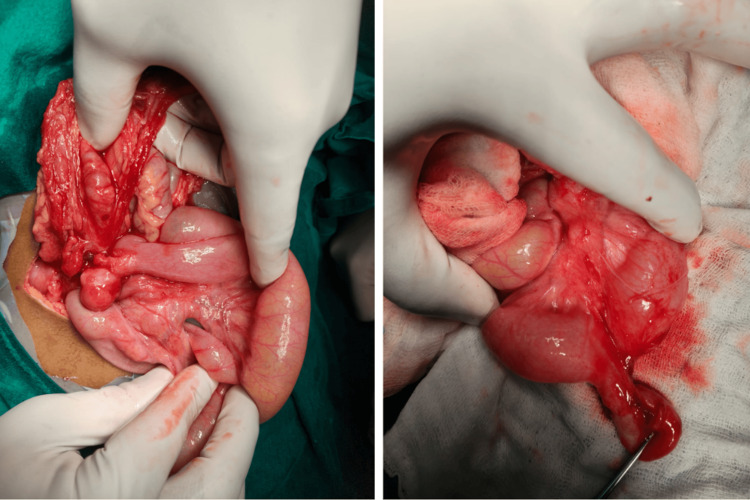
Intraoperative findings (A) Wide view showing the perforated Meckel's diverticulum adherent to the ascending colon; (B) Close-up of the inflamed diverticular structure and associated fibrinous adhesions

Based on the clinical presentation and radiological evidence of a contained perforation, the patient was prepared for emergency exploratory laparotomy. Under general anesthesia, a supraumbilical transverse incision was made. Upon entering the peritoneal cavity, clumped bowel loops were noted in the right hypochondrium, densely adherent to both the liver and the ascending colon. Following complete mobilization, a Meckel’s diverticulum was identified approximately 40 cm proximal to the ileocecal valve (Figure 1). The diverticulum exhibited a perforation at its tip and was firmly adherent to the ascending colon, resulting in significant luminal obstruction. A segmental resection of the involved ascending colon was performed, followed by a side-to-side ileocolic anastomosis using 4-0 polyglactin sutures. To restore small bowel continuity after diverticular excision, a separate primary ileal anastomosis was completed, resulting in a double anastomosis. Hemostasis was achieved, and the abdomen was closed in layers. Postoperatively, the patient received broad-spectrum antibiotics (ceftriaxone) and analgesics. The recovery was uneventful; the patient tolerated oral intake by the third postoperative day and remained hemodynamically stable throughout. He was discharged on the seventh postoperative day with oral antibiotics and nutritional supplementation.

Histopathology findings

**Figure 2:**
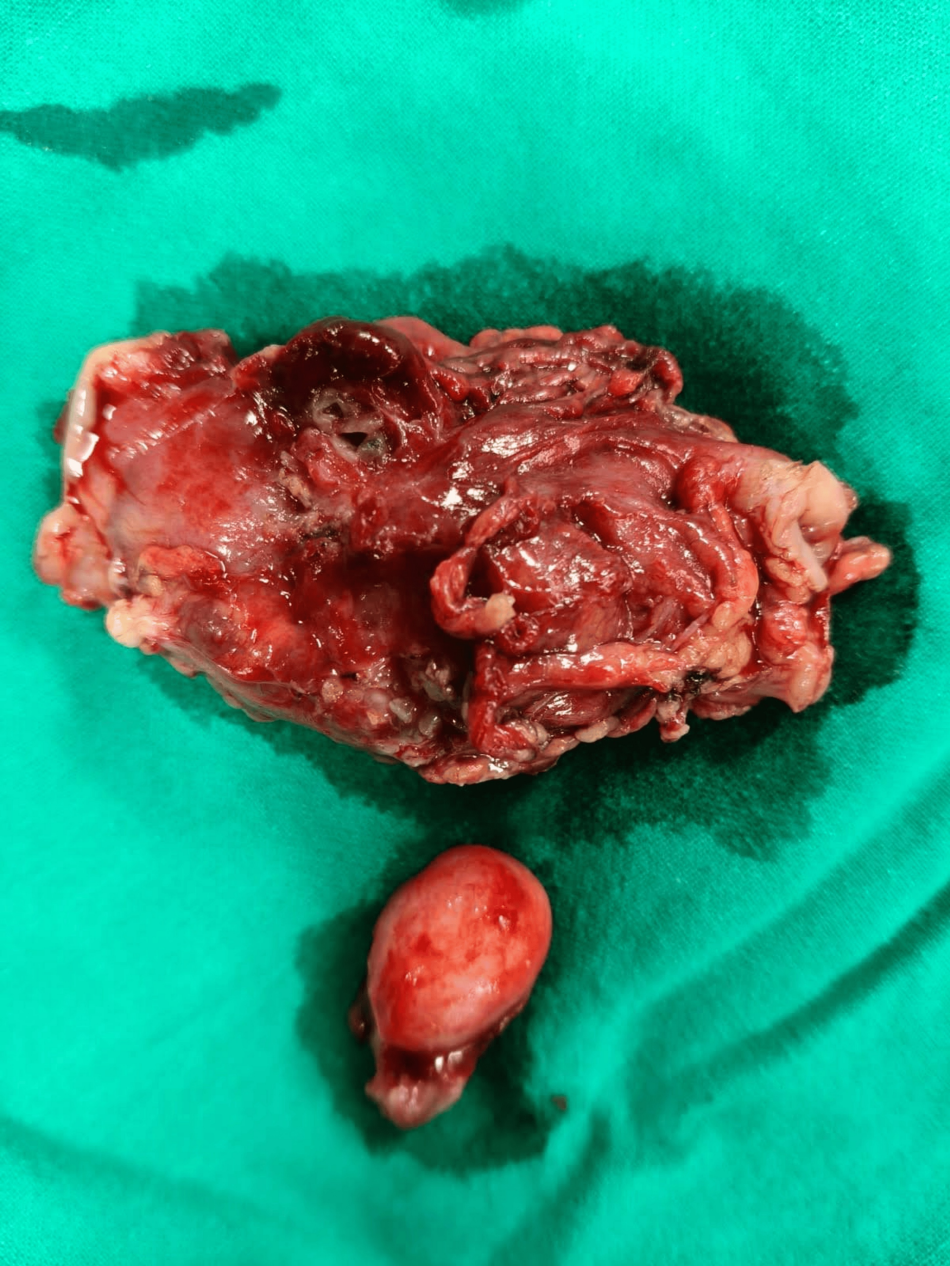
Surgically resected specimen of perforated Meckel's diverticulum

Following surgical resection, the excised specimens were subjected to histopathological analysis, which provided the definitive diagnosis. Gross examination revealed a tubular diverticular pouch measuring 2.9 × 1.5 × 1.0 cm with a congested external surface and a lumen obliterated by a fecolith. The second segment consisted of a portion of the ascending colon measuring 4.5 × 3.5 × 2.5 cm with a congested, dull-appearing serosa (Figure 2).

**Figure 3:**
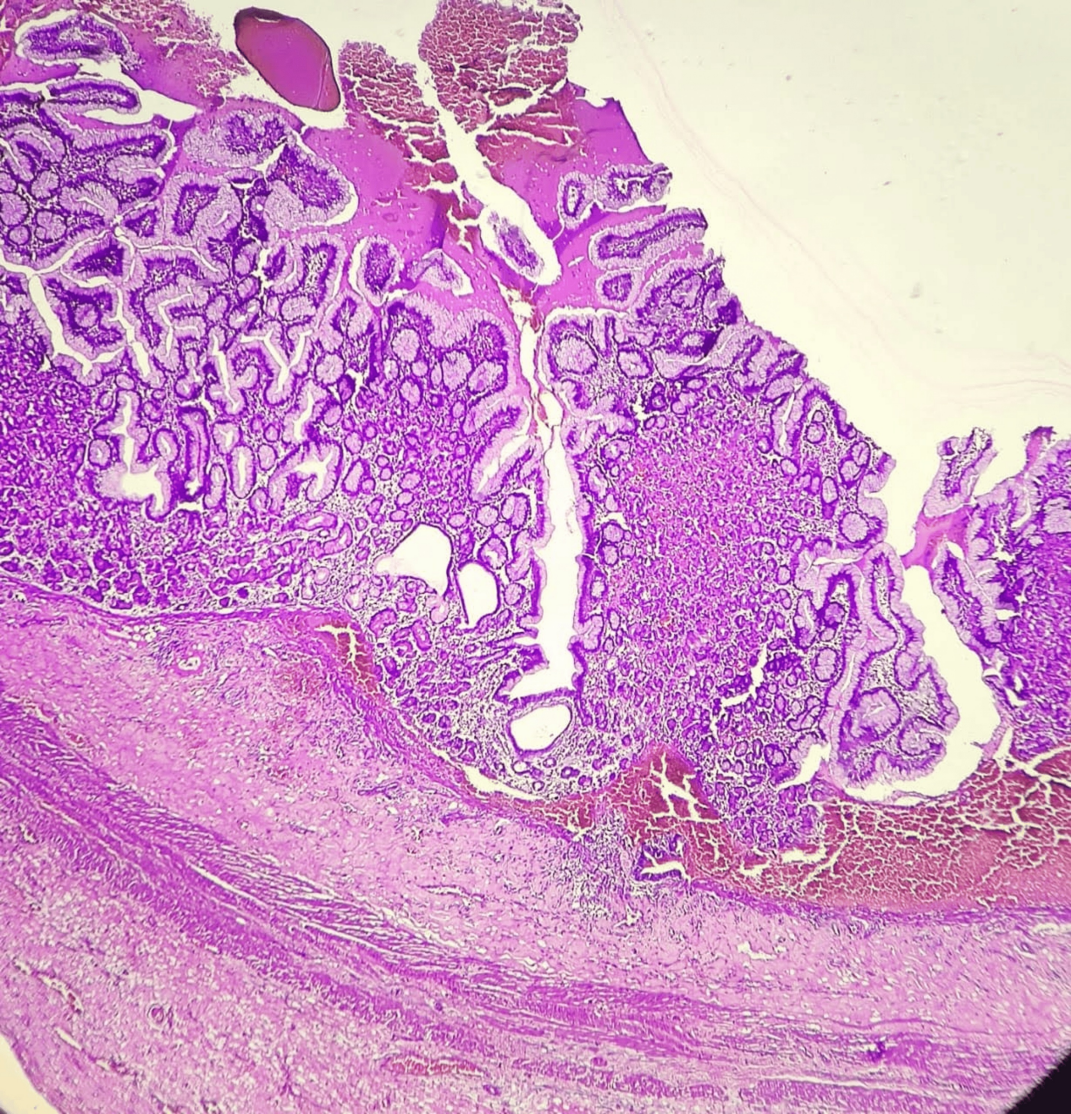
Microscopy showing ectopic gastric mucosa [H and E, 100x]

**Figure 4:**
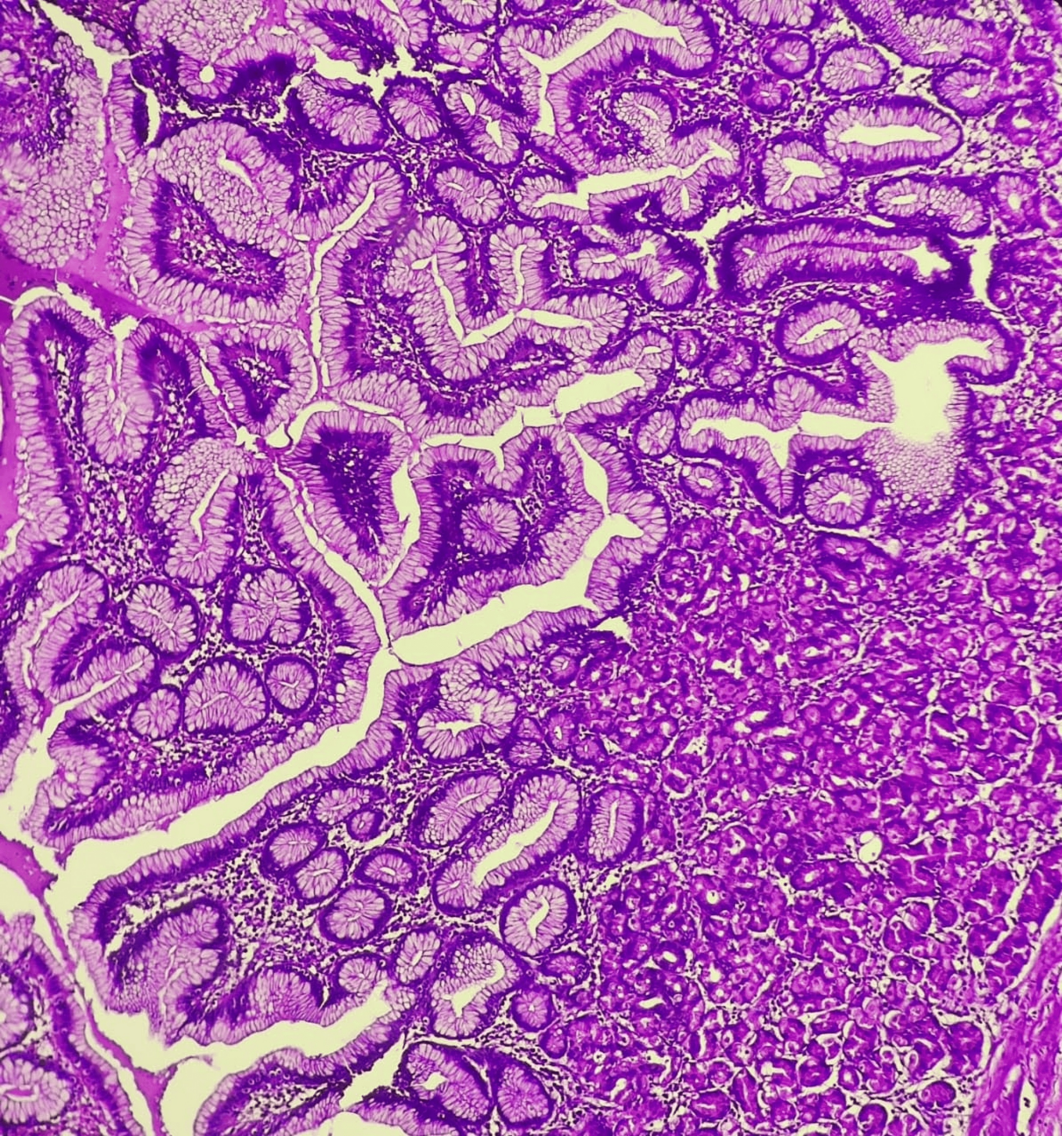
Microscopy of ectopic gastric mucosa lined by foveolar epithelium and showing specialized parietal and oxyntic glands [H and E, 400X]

**Figure 5:**
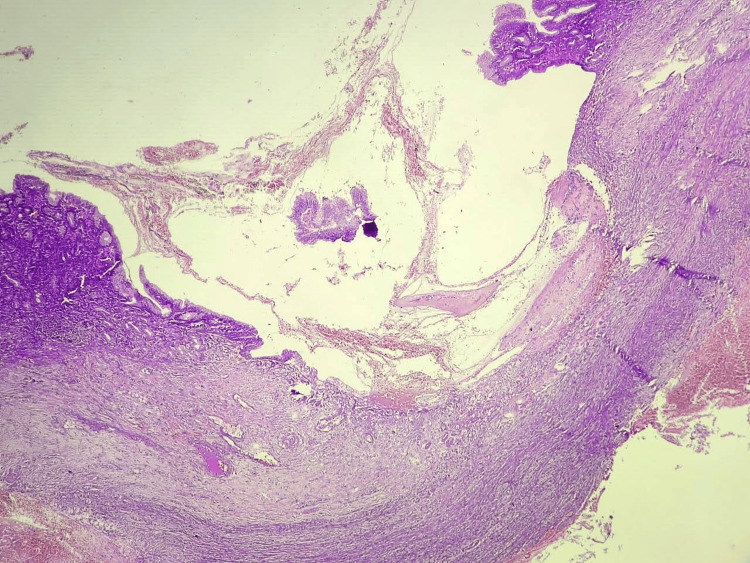
Microscopy of gastric ulcer [H and E, 100x]

**Figure 6:**
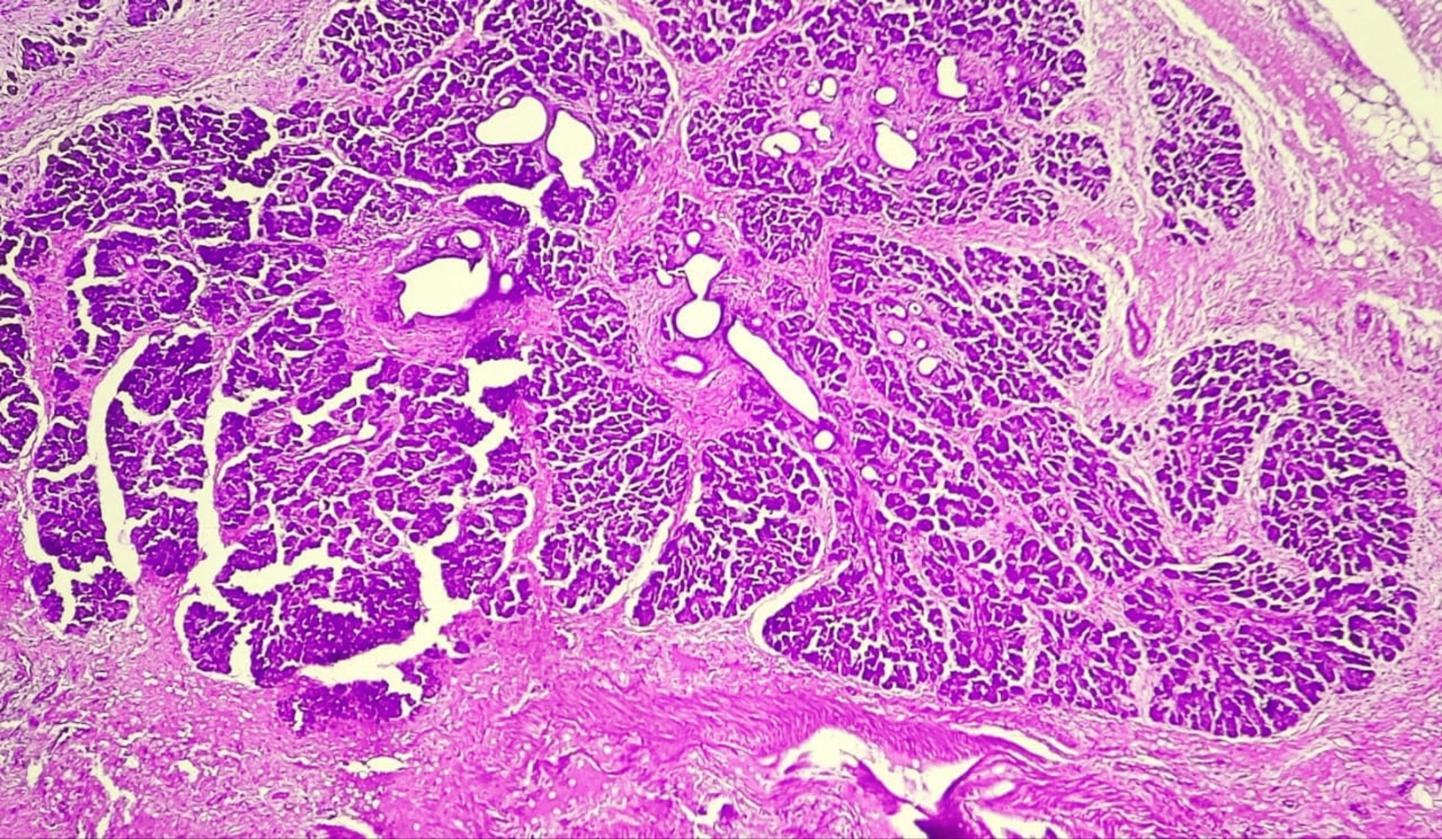
Microscopy of ectopic pancreatic tissue showing well-defined acini and islets [H and E, 100x]

Microscopic examination of the resected Meckel's diverticulum confirmed the presence of dual ectopic tissue and the site of perforation. Sections of the diverticulum revealed heterotopic gastric mucosa, characterized by a mucosal lining of foveolar epithelium and a lamina propria containing both parietal and oxyntic glands (Figures 3, 4). Adjacent to this gastric mucosa, a focal, well-defined peptic ulcer was identified (Figure 5). Furthermore, a distinct focus of ectopic pancreatic tissue was observed, featuring clear pancreatic acini and islets (Figure 6).

**Figure 7:**
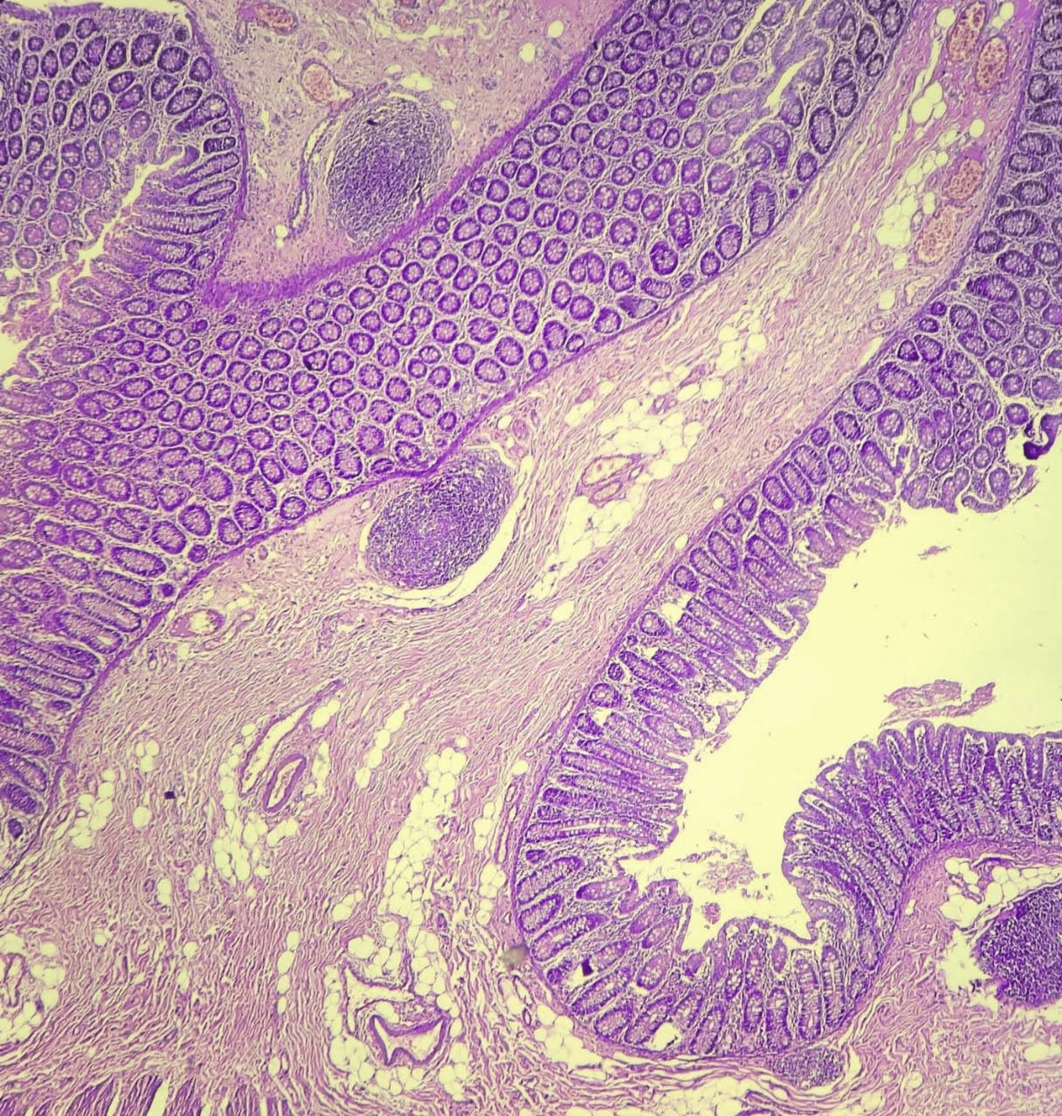
Microscopy of ascending colon wall with lymphoid follicles [H and E, 100x]

**Figure 8:**
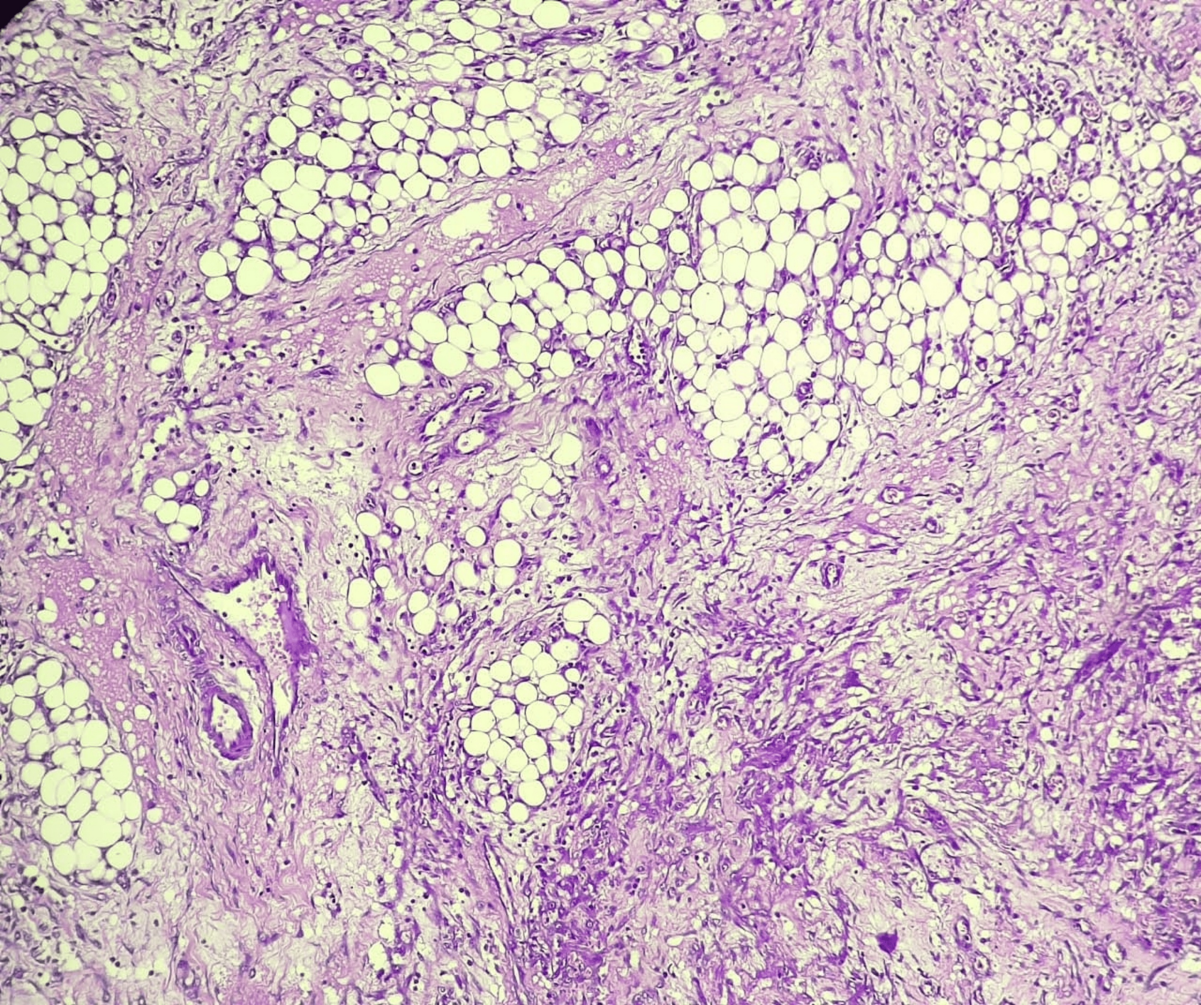
Microscopy showing features of mesenteritis surrounding ascending colon [H and E, 100X]

The site of perforation was marked by transmural necrosis and granulation tissue. The serosa exhibited dilated, congested vessels, hemorrhage, and a covering of fibrinosuppurative exudate. Sections from the adjacent ascending colon showed chronic non-specific colitis with serositis, characterized by chronic inflammatory infiltrates, lymphoid follicles, submucosal edema, and mesenteric granulation tissue (Figures 7, 8). These histopathological features confirmed a perforated Meckel’s diverticulum with dual gastric and pancreatic heterotopia and associated reactive chronic colitis.

## Discussion

Meckel's diverticulum (MD) is the most common congenital anomaly of the gastrointestinal tract, with an incidence of 1-2% and an overall complication risk of 4-6% [1-3]. Although most MD cases remain asymptomatic and are incidentally discovered during surgery, complications such as perforation are exceedingly rare, accounting for less than 0.5% of symptomatic cases [6]. This rarity, combined with a clinical presentation that closely mimics acute appendicitis, especially in pediatric patients, poses a significant diagnostic challenge.

Our case is notable for several atypical features, primarily the presence of dual heterotopic tissue (both gastric and pancreatic). Gastric mucosa, found in approximately 52% of symptomatic pediatric MD cases, secretes hydrochloric acid and pepsin, which can lead to mucosal ulceration. Pancreatic heterotopia, present in only about 5% of cases, further secretes proteolytic enzymes such as trypsin, which likely exacerbate mucosal injury [7]. The synergistic effect of these tissues accelerates ulceration and precipitates perforation, underlining the increased risk of complications associated with dual heterotopia.

Preoperative diagnosis of complicated MD remains difficult due to non-specific symptoms and imaging features; historically, only 10% of cases are diagnosed preoperatively [5]. Conventional imaging modalities like ultrasound and CT scans often fail to distinguish MD from normal bowel loops, especially in emergency settings. Although technetium-99m pertechnetate scintigraphy (Meckel's scan) has high specificity for detecting ectopic gastric mucosa, its sensitivity is variable, and it is often unavailable in resource-limited emergency settings [1]. In our patient, contrast-enhanced CT (CECT) provided critical preoperative evidence by identifying the inflamed diverticulum, adjacent inflammatory changes, and a contained perforation, thereby aiding surgical planning.

However, surgical resection and histopathological examination remain the gold standard for symptomatic or complicated MD. While a simple diverticulectomy or wedge resection is often preferred, the choice of procedure must be tailored to the width of the diverticular base and the extent of local inflammation [4]. In our patient, the presence of a broad base and significant surrounding inflammation necessitated a segmental ileal resection with primary anastomosis. Furthermore, because the diverticulum was densely adherent to the ascending colon, a segmental colectomy with ileocolic anastomosis was required. Although conservative adhesiolysis is an option in less severe cases, the degree of transmural necrosis and the presence of a dense inflammation involving the colonic wall mandated this more extensive approach. This decision was critical to mitigate the risks of postoperative leaks or persistent obstruction from the compromised tissue and ensure a safe anastomosis.

## Conclusions

This case highlights the importance of including Meckel's diverticulum with rare complications in the differential diagnosis of the pediatric acute abdomen, particularly when clinical features mimic acute appendicitis. The presence of dual gastric and pancreatic heterotopia significantly increases the life-threatening potential of this congenital anomaly, as dual heterotopic tissues are thought to create a synergistic effect combining acid secretion with proteolytic enzymes that likely precipitate early perforation, as seen in this case. While the clinical presentation is often ambiguous, a high index of clinical suspicion combined with the judicious use of contrast-enhanced CT is crucial for timely preoperative recognition. Ultimately, histopathological analysis remains the gold standard for confirming the diagnosis and characterizing the heterotopic tissue. This report contributes to the limited literature on this rare presentation and reinforces the necessity of a systematic, resource-appropriate diagnostic and surgical pathway to optimize outcomes in pediatric patients with an atypical acute abdomen.
